# Best Practice Recommendations for the Diagnosis and Management of Children With Pediatric Inflammatory Multisystem Syndrome Temporally Associated With SARS-CoV-2 (PIMS-TS; Multisystem Inflammatory Syndrome in Children, MIS-C) in Switzerland

**DOI:** 10.3389/fped.2021.667507

**Published:** 2021-05-26

**Authors:** Luregn J. Schlapbach, Maya C. Andre, Serge Grazioli, Nina Schöbi, Nicole Ritz, Christoph Aebi, Philipp Agyeman, Manuela Albisetti, Douggl G. N. Bailey, Christoph Berger, Géraldine Blanchard-Rohner, Sabrina Bressieux-Degueldre, Michael Hofer, Arnaud G. L'Huillier, Mark Marston, Patrick M. Meyer Sauteur, Jana Pachlopnik Schmid, Marie-Helene Perez, Bjarte Rogdo, Johannes Trück, Andreas Woerner, Daniela Wütz, Petra Zimmermann, Michael Levin, Elizabeth Whittaker, Peter C. Rimensberger, the PIMS-TS working group of the Interest Group for Pediatric Neonatal Intensive Care (IGPNI) of the Swiss Society of Intensive Care and the Pediatric Infectious Diseases Group Switzerland (PIGS)

**Affiliations:** ^1^Pediatric and Neonatal Intensive Care Unit, Children's Research Center, University Children's Hospital Zurich, Zurich, Switzerland; ^2^Paediatric Intensive Care Unit, Child Health Research Centre, Queensland Children's Hospital, The University of Queensland, Brisbane, QLD, Australia; ^3^Division of Respiratory and Critical Care Medicine, University of Basel Children's Hospital, Basel, Switzerland; ^4^Department of Pediatric Hematology and Oncology, University Children's Hospital, Eberhard Karls University, Tübingen, Germany; ^5^Division of Neonatal and Pediatric Intensive Care, Department of Child, Woman, and Adolescent Medicine, Faculty of Medicine, Geneva University Hospitals, Geneva, Switzerland; ^6^Department of Pediatrics, Inselspital, Bern University Hospital, University of Bern, Bern, Switzerland; ^7^Alder Hey Children's Hospital, National Health System Foundation Trust, Liverpool, United Kingdom; ^8^Department of Infectiology and Vaccinology, University Children's Hospital Basel, Basel, Switzerland; ^9^Department of Haematology, Children's Research Center, University Children's Hospital Zurich, Zurich, Switzerland; ^10^Pediatric and Neonatal Intensive Care Unit, Children's Hospital St. Gallen, St. Gallen, Switzerland; ^11^Division of Infectious Diseases, Hospital Epidemiology, University Children's Hospital Zurich, Zurich, Switzerland; ^12^Unit of Immunology and Vaccinology, Division of General Pediatrics, Department of Pediatrics, Gynecology and Obstetrics, Geneva University Hospitals, University of Geneva, Geneva, Switzerland; ^13^Pediatric Cardiology Unit, University Hospital Lausanne, Lausanne, Switzerland; ^14^Pediatric Immuno-Rheumatology of Western Switzerland, Department Women-Mother-Child, Lausanne University Hospital, Lausanne, and University Hospitals of Geneva, Geneva, Switzerland; ^15^Division of Immunology, Children's Research Center, University Children's Hospital Zurich, Zurich, Switzerland; ^16^Pediatric Intensive Care Unit, University Hospital Lausanne, Lausanne, Switzerland; ^17^Department of Rheumatology, University Children's Hospital Basel, Basel, Switzerland; ^18^Division of Pediatric Cardiology, Pediatric Heart Center, University Children's Hospital Zurich, Zurich, Switzerland; ^19^Department of Paediatrics, Faculty of Science and Medicine, Fribourg Hospital, University of Fribourg, Fribourg, Switzerland; ^20^Infectious Diseases Research Group, Murdoch Children's Research Institute, Parkville, VIC, Australia; ^21^Section of Paediatric Infectious Diseases, Imperial College London, London, United Kingdom; ^22^Paediatric Infectious Diseases, Imperial College Healthcare National Health System Trust, London, United Kingdom

**Keywords:** child, COVID-19, Kawasaki disease, multisystem inflammatory syndrome, MIS-C, pediatric inflammatory multisystem syndrome, septic shock

## Abstract

**Background:** Following the spread of the coronavirus disease 2019 (COVID-19) pandemic a new disease entity emerged, defined as Pediatric Inflammatory Multisystem Syndrome temporally associated with COVID-19 (PIMS-TS), or Multisystem Inflammatory Syndrome in Children (MIS-C). In the absence of trials, evidence for treatment remains scarce.

**Purpose:** To develop best practice recommendations for the diagnosis and treatment of children with PIMS-TS in Switzerland. It is acknowledged that the field is changing rapidly, and regular revisions in the coming months are pre-planned as evidence is increasing.

**Methods:** Consensus guidelines for best practice were established by a multidisciplinary group of Swiss pediatric clinicians with expertise in intensive care, immunology/rheumatology, infectious diseases, hematology, and cardiology. Subsequent to literature review, four working groups established draft recommendations which were subsequently adapted in a modified Delphi process. Recommendations had to reach >80% agreement for acceptance.

**Results:** The group achieved agreement on 26 recommendations, which specify diagnostic approaches and interventions across anti-inflammatory, anti-infectious, and support therapies, and follow-up for children with suspected PIMS-TS. A management algorithm was derived to guide treatment depending on the phenotype of presentation, categorized into PIMS-TS with (a) shock, (b) Kawasaki-disease like, and (c) undifferentiated inflammatory presentation.

**Conclusion:** Available literature on PIMS-TS is limited to retrospective or prospective observational studies. Informed by these cohort studies and indirect evidence from other inflammatory conditions in children and adults, as well as guidelines from international health authorities, the Swiss PIMS-TS recommendations represent best practice guidelines based on currently available knowledge to standardize treatment of children with suspected PIMS-TS. Given the absence of high-grade evidence, regular updates of the recommendations will be warranted, and participation of patients in trials should be encouraged.

## Introduction

Subsequent to the first wave of the coronavirus disease 2019 (COVID-19) pandemic (1), clusters of children presenting with unusual multisystem inflammatory conditions emerged (2, 3). The clinical syndrome showed some heterogeneity with patients presenting either akin to toxic shock, Kawasaki-disease like or with undifferentiated inflammatory characteristics, in addition to evidence of current or past COVID-19 infection in the majority of cases (4, 5). Accordingly, this new disease entity has been called Pediatric Inflammatory Multisystem Syndrome temporally associated with COVID-19 (PIMS-TS) as per the Royal College of Pediatrics and Child Health case definition (RCPCH) (6) and Multisystem Inflammatory Syndrome associated with COVID-19 (MIS-C) as per the Center for Disease Control (CDC) (7) and the WHO (World Health Organisation) (8).

While a number of studies have attempted to explain the underlying biological and genetic mechanisms of PIMS-TS, its pathophysiology and the observed variation in clinical presentation remains largely unknown (9–11). It is currently assumed that a complex process involving SARS-CoV-2-specific antigen presentation to autoreactive T cells, superantigen-like viral structures, cross-reactive SARS-CoV-2-specific antibodies and unbalanced cytokine responses may initiate PIMS-TS in genetically predisposed children (12–16). In other pediatric hyperinflammatory syndromes, such as Kawasaki disease (KD), macrophage activation syndrome (MAS), and hemophagocytic lymphohistiocytosis (HLH), timely initiation of anti-inflammatory therapy and immunosuppression can often reverse the hyper-inflammatory state and prevent or mitigate organ damage. Therefore, therapeutic immune modulation is widely used as a mainstay of PIMS-TS treatment with the aim to inhibit cytokine secretion and restore immune homeostasis. In addition, PIMS-TS patients may also show signs of vasculitis, endothelial damage, and thrombosis, hence antiplatelet and anticoagulation management in PIMS-TS represent important additional considerations (17).

Cardiac involvement includes a supposed post-infectious myocarditis or myocardial injury and the development of coronary artery abnormalities with a minority developing coronary artery abnormalities. Myocardial injury is thought to be attributed to an acute and dysregulated immune response related to cytokine storm, endothelial damage, microvascular dysfunction, and ischemic injury (18). The tropism of SARS-Cov-2 to myocytes and endothelial cells with the facilitated entry by binding to the angiotensin-converting enzyme 2 receptors may contribute to the high percentage of cardiac involvement (19).

In order to standardize management in Switzerland, we aimed to develop best practice recommendations for the diagnosis and treatment of children with PIMS-TS.

## Statement of Uncertainty

At present, professionals remain confronted with substantial uncertainties regarding clinical phenotypes, long-term outcomes, and optimal management (20). In the absence of randomized trials, evidence for best treatment is minimal for the diagnostic, anti-inflammatory, anti-infectious, and supportive measures which have been proposed (9, 21). Recommendations therefore are based primarily on expert opinion and similar recommendations in the United Kingdom. The field is rapidly changing with reports being published on an almost weekly basis (22, 23), hence revision and updates of the recommendations will be required regularly.

## Methods

Subsequent to a call for Expressions of Interest, the Interest Group for Pediatric and Neonatal Intensive Care (IGPNI) of the Swiss Society of Intensive Care Medicine (SSICM) and the Pediatric Infectious Diseases Group Switzerland (PIGS) formed a working group on PIMS-TS. In total, 24 panelists across the fields of pediatric intensive care, infectious diseases, immunology and rheumatology, hematology, cardiology, and nursing composed the panel.

Four subgroups focused on the domains of (i) disease criteria and diagnosis, (ii) anti-inflammatory therapies, (iii) anti-infective therapies, and (iv) additional support therapies including coagulation management. Each subgroup performed a focused literature review on publications since the description of PIMS-TS in early 2020 until December 2020. In addition, we searched for available pathways, diagnostic, therapeutic, and follow-up recommendations from different international institutions (24–27). Over a period of 5 weeks, weekly virtual meetings of the entire working group were held to develop and discuss the recommendations in a modified Delphi process. Finally, voting was performed by the entire panel for each recommendation using Survey Monkey. The threshold for recommendations was met if >80% of panelists voted for full agreement on an item. The thereby developed guideline was piloted during 4 weeks in January 2021, subsequent to which an update and re-voting occurred on items which were agreed to be changed in the panel.

## Recommendations

### Diagnosis

1. For PIMS-TS, the following case definition should be used:
2. Adapted RCPCH—case definition (6, 28)
  - Patient aged <18 years of age. Persistent fever + inflammation (elevated CRP and neutrophils, or lymphopaenia) + single or multiorgan dysfunction (shock, cardiac, respiratory, renal, gastrointestinal, neurological) + additional features (see Table 1). This may include children fulfilling full or partial criteria for Kawasaki disease (KD).
  - Exclusion of any other probable cause, such as bacterial sepsis, staphylococcal/streptococcal shock syndromes, and viral infections associated with myocarditis. Waiting for these results should not delay seeking expert advice.
  - Positive for current or recent SARS-CoV-2 infection by PCR, serology, or antigen test; or COVID-19 exposure within 4 weeks prior to the onset of symptoms. Waiting for these results should not delay seeking expert advice.
3. If a child fulfills the diagnostic criteria for PIMS-TS we recommend classifying these patients according to the presenting phenotype due to the implications for diagnostic workup and management:
  - *Shock-like presentation*: signs of shock as per the Goldstein 2005 definition of cardiovascular failure (29) (Appendix)
  - *Kawasaki disease-like presentation*: complete or incomplete with cardiac involvement, according to AHA (30)
  - *Undefined inflammatory presentation*: persistent pyrexia with signs of PIMS-TS but not meeting shock criteria nor having cardiac involvement.

**Table 1:** List of diagnostic criteria for PIMS-TS.

**General**	**Criteria**	
**Clinical features**		
Required	Fever	O
Organ systems	Single or multi-organ involvement	
Gastrointestinal	Abdominal pain, diarrhea, vomiting	O
	Abnormal liver function tests	O
	Colitis, ileitis, ascites	O
Cardiovascular	Hypotension, shock, oliguria	O
	Myocardial dysfunction, pericardial effusion	O
	Coronary artery abnormalities	O
Respiratory	Cough, sore throat	O
	Oxygen requirement	O
	Patchy infiltrates, pleural effusion	O
Dermatologic	Conjunctivitis, periorbital swelling/redness	O
	Mucus membrane changes	O
	Rash	O
	Lymphadenopathy	O
	Swollen hands and feet	O
Neurologic	Headache, confusion, irritability, reduced level of consciousness	O
	Syncope	O
**Abnormal laboratory findings indicating inflammation (any combination)**		
Inflammatory markers	Elevated CRP/fibrinogen/D-Dimers/ferritin, hypoalbuminemia, lymphopaenia, neutrophilia	O
Cardiac biomarkers	Elevated Troponin T/NT-pro-BNP	O
COVID-19 contact	Either confirmed or putative	O
Confirmed	Positive for current or recent SARS-CoV-2 infection by PCR, serology, or antigen test	O
Putative	COVID-19 exposure within the 4 weeks prior to the onset of symptoms	O
**No alternative plausible diagnosis (microbial or inflammatory)**		O

*Patients must be below 18 years and meet at least one criterion for each group, including (i) presence of fever, (ii) organ involvement, (iii) laboratory evidence of inflammation, (iv) microbiologically proven or putative COVID-19 contact, and (v) exclusion of other causes*.

Overall, PIMS-TS remains a rare condition and most children with SARS-CoV-2 infection will remain asymptomatic or will exhibit only mild symptoms (4, 31). Predominant clinical features include persistent fever and gastrointestinal symptoms, e.g., abdominal pain, vomiting or diarrhea. Many patients may exhibit additional clinical features (Table 1). Cardiovascular impairment can be manifest at presentation or develop during admission (18, 32). Coronary artery aneurysms (CAA) may develop post-discharge up to 3 weeks after disease onset (30). Some patients show rapid clinical deterioration often characterized by a vasoplegic shock state requiring admission to intermediate or intensive care units (IMC/PICU) (4, 33).

It is paramount to avoid anchoring bias given that PIMS-TS remains a rare condition (34), and in view of the fact that many children suffering from other disease during the pandemic may have concomitant microbiological evidence of SARS-CoV-2 exposure. Other, more common differential diagnoses need to be considered, such as but not restricted to:

– Invasive bacterial infection
– Sepsis
– Toxic shock syndrome (TSS)
– Staphylococcal Scalded Skin Syndrome (SSSS)
– Kawasaki disease (KD)
– Viral myocarditis/infection (such as EBV, CMV, adenovirus, enterovirus, and other viruses)
– Serum sickness
– Acute appendicitis/acute surgical abdomen
– Gastroenteritis
– Macrophage activation syndrome (MAS) and hemophagocytic lymphohistiocytosis (HLH) (Appendix)
– Malignant diseases, e.g., acute leukemia.

As pediatric Emergency Departments face large numbers of febrile children, we recommend a staged diagnostic approach, starting with standard investigations for all children where PIMS-TS is suspected (Table 2), followed by more in-depth diagnostic work-up in children with evidence of severe disease, or those with diagnostic uncertainty.

**Table 2:** Recommendations for diagnostic work-up in children evaluated for PIMS-TS.

**Baseline investigations in a febrile child with features of PIMS-TS** (according to disease severity, for a child in shock baseline and supplementary investigations should be carried out at the same time)	Full blood count (FBC) C-reactive protein (CRP) Urea, creatinine, electrolytes (U&E) Liver function tests (LFTs) In addition, as clinically indicated:– Blood cultures (always before starting antibiotics)– Urine microscopy and culture– Lumbar puncture, if no contraindication present– NPA: respiratory panel, SARS-CoV-2 PCR
**Supplementary investigations in case of suspected PIMS-TS** (in addition to baseline investigations)	Blood gas, lactate, glucose Erythrocyte sedimentation rate (ESR) Coagulation: INR, aPTT, Fibrinogen D-dimers Ferritin Albumin Troponin T NT-pro-BNP LDH CK, and CK-MB SARS-CoV-2 serology Store serum (always before starting IVIG) Store EDTA 12-lead ECG and echocardiography Chest radiograph Abdominal ultrasound (if gastrointestinal symptoms)
**Other optional extended investigations** (according to clinical presentation after MDT discussion. Those should NOT delay seeking expert opinion or treatment)	EBV/CMV/Adeno-/Enterovirus blood PCR and consider other viral PCRs Stool virology/microbiology IL-10, IL-6 Triglycerides sCD25^*^ ^*^consider full HLH screen if suggestive features present (e.g., splenomegaly, fibrinogen normal or low; ferritin >2,000 μg/l): Perforin-, SAP-, and XIAP-expression, NK cell degranulation and consider HLH-directed therapy (MDT)
**Repeat investigations:**	*Critical patient (deteriorating and/or in PICU):* Approx. 24 hourly: FBC, CRP, U&E, LFTs, coagulation, ferritin Other parameters guided by clinical progress Cardiac biomarkers, echocardiography in consultation with cardiology, ECG *Non-critical patient with ongoing pyrexia:* 24–48 hourly: FBC, CRP U&E, LFTs, ferritin ^*^as above Echocardiography 48 hourly (in consultation with cardiology) *Child improving ± defervescence:* 48 hourly or pre-discharge: FBC, CRP, U&E, LFTs, ESR Consultation with cardiology prior to discharge

*Where possible, PIMS-TS patients should be enrolled in observational or interventional studies, which may include additional diagnostics*.

* *Children with evidence of cardiac involvement should be discussed with a tertiary center for cardiology involvement and care in PICU should be considered*.

PIMS-TS may manifest with features of classic or incomplete (atypical) KD with, or without cardiac involvement (30). Although the clinical presentation of PIMS-TS patients shares similarities with KD and TSS (4, 31), there are several differences in the clinical presentation compared to classical KD and patients with PIMS-TS by definition lack microbial confirmation of a staphylococcal or streptococcal infection. Compared to classical KD, patients with PIMS-TS more often present with prominent gastrointestinal and neurologic symptoms (18), and the incidence of cardiac involvement is even higher in PIMS-TS patients. More than 50% of children with PIMS-TS do develop some sort of cardiac involvement, defined by elevation of cardiac biomarkers, systolic or diastolic myocardial dysfunction or even shock. In addition, there is limited evidence that conduction abnormalities are more common in PIMS-TS, including heart block (35, 36).

Early involvement of a pediatric multidisciplinary team (MDT) including intensive care, immunology, infectious diseases, rheumatology, cardiology, hematology, and others (e.g., general surgery) should be considered. In addition to cardiac biomarkers (such as cTnT and NT-pro-BNP) the cardiac evaluation of a patient with PIMS-TS should include a 12-lead electrocardiogram and echocardiography (Table 2). Diagnostic measures, including laboratory tests such as markers of inflammation and organ dysfunction, and imaging modalities such as transthoracic Doppler 2D echocardiography should be repeated sequentially depending on the presentation, disease severity, and evolution to guide escalation and de-escalation of therapy, and to rule out other diagnoses.

A substantial proportion of children with PIMS-TS initially manifest significantly raised levels of NT-pro-BNP and modest elevations of cardiac troponin, which sometimes take days to weeks to normalize (19, 32). In patients with preserved ejection fraction, increased cardiac biomarkers likely reflect subclinical myocardial injury and may be associated with more subtle changes in diastolic function (37). Commonly observed echocardiographic findings include depressed systolic LV and RV function. Moreover, mitral regurgitation, and mild pericardial effusion have been described. While most patients show rapid improvement in LV systolic function and even normalization within 1–2 weeks after initial presentation, diastolic dysfunction often persists until early follow up or longer (37).

ECG registration is recommended at the time of diagnosis and every 48 h while inpatient, daily in patients on PICU. Most ECG findings are non-specific and include ST-segment changes, QTc prolongation and premature atrial or ventricular beats. Another common finding is first-degree (rarely higher grade) atrioventricular block—which may progress after initial diagnosis (35, 36). Single cases of atrial fibrillation or sustained arrhythmias leading to hemodynamic collapse have been described (38). All inpatients should be monitored by telemetry. In well patients with unremarkable ECG recording, oxygen saturation and heart rate monitoring is deemed to be sufficient. In outpatients with first-degree atrioventricular block or arrhythmias Holter monitoring is recommended (27).

Coronary artery aneurysms represent a serious complication and may place patients at risk for coronary artery thrombosis or stenosis, myocardial infarction and cardiac death. Coronary artery abnormalities have been reported to appear in 8–24% of patients with PIMS-TS irrespective of the phenotype (18, 32). Most of them are dilation or small aneurysms, but large/giant aneurysms may occur (38), even during the convalescent period (4, 39). While, coronary artery dilation resolve within 4–8 weeks without sequelae, aneurysms may lead to coronary artery stenosis and thrombosis even when they reduce in size over weeks to months (30).

### Anti-inflammatory Therapies

3. Therapeutic immune-modulation in PIMS-TS patients requires a multidisciplinary approach. Clinicians should re-evaluate the patient response and consider differential diagnoses at every step.

**Figure 1:**
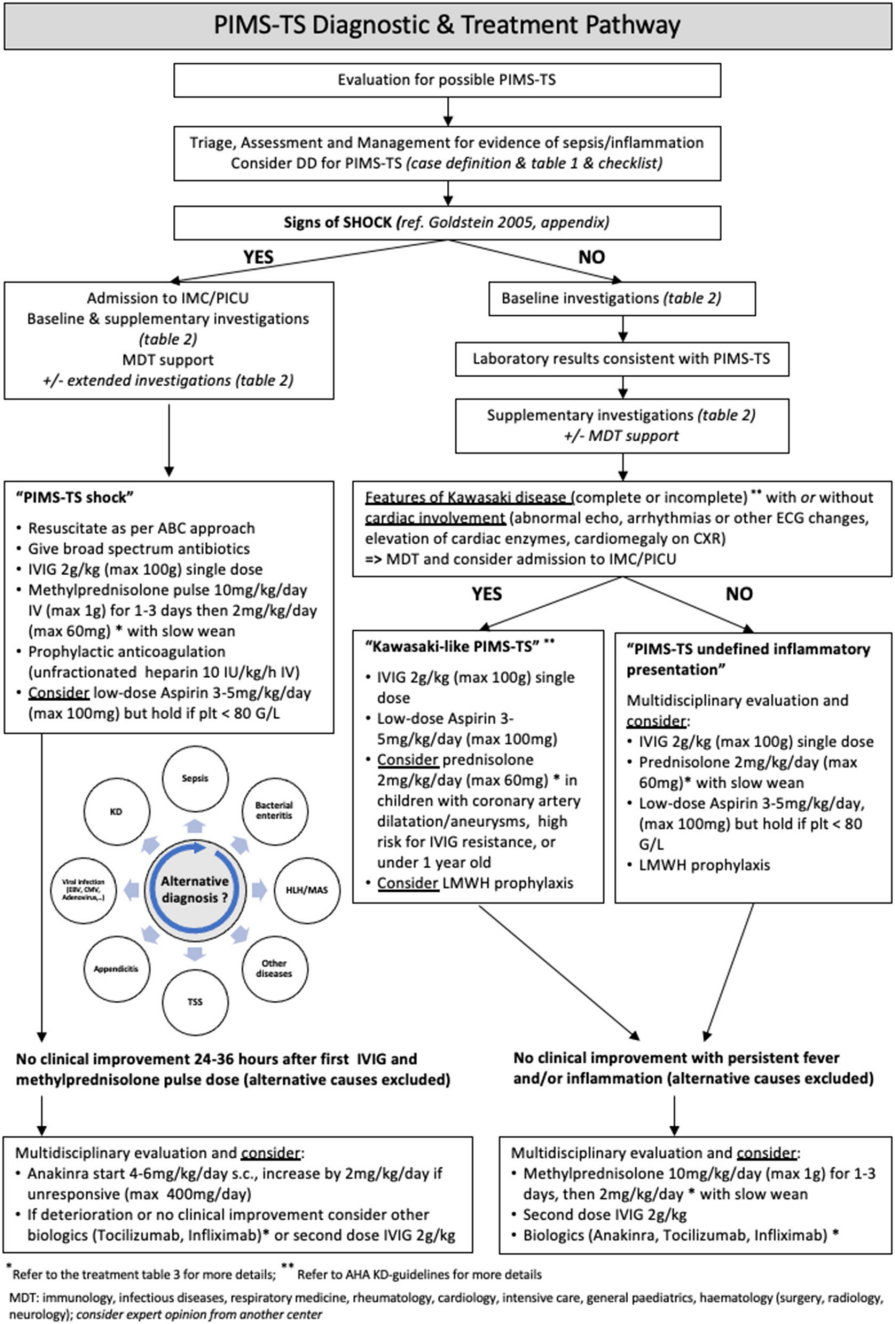
PIMS-TS diagnostic and therapeutic algorithm.

We recommend a MDT approach to guide initiation, escalation, and tapering of empiric immunomodulatory therapy, particularly because available evidence for such treatment is currently only based on observational reports. The panel recommends using a management algorithm (Figure 1) to guide the step-wise selection of the initial interventions. Dosing recommendations are provided in Table 3 and were adapted from the Imperial College Healthcare NHS Trust PIMS-TS guideline (Prof. Elizabeth Whittaker, personal communication).

4. In patients with Kawasaki disease-like PIMS-TS, immunomodulation and management should follow established guidelines for Kawasaki disease.
5. In patients with PIMS-TS shock, we recommend using immunoglobulins (IVIG, 2 g/kg).
6. In patients with PIMS-TS shock we recommend treatment with intravenous pulse high-dose methylprednisolone (10 mg/kg q24 h for 1–3 days, max. 1 g/day).
7. In non-shocked patients with PIMS-TS undefined inflammatory presentation clinicians should consider administration of immunoglobulins (IVIG, 2 g/kg).
8. In non-shocked patients with PIMS-TS (Kawasaki-like presentation or undefined inflammatory presentation) clinicians should consider administration of prednisolone (2 mg/kg q24 h, max. 60 mg/day).
9. In all patients with confirmed PIMS-TS treated with steroids, steroids should be tapered over a period of 2–6 weeks depending on the clinical course and considering the clinical and biochemical (such as CRP, D-Dimer, and ferritin levels) response.

**Table 3:** Anti-inflammatory therapies in patients with PIMS-TS.

**Class**	**Drug**	**Route**	**Dose**	**Duration**	**Comments and side effects**
Blood products	**IVIG**	IV	2 g/kg (max 100 g)	Infusion over 12 h	*Side effects:* Aseptic meningitis, volume load, systemic inflammation, hemolytic anemia, neutropenia. *Slower the rate or divide the dose over 2 days if signs of volume overload or severe cardiac dysfunction*
Corticosteroids	**Methylprednisolone**	IV	2 mg/kg daily (max 60 mg/day) or 10 mg/kg daily for 1–3 days (max 1 g/day)	1–3 days discuss in MDT	*Side effects:* Hyperglycemia, hypertension, agitation Note: dose increase up to 30 mg/kg can be considered if refractory
	**Prednisolone**	PO	1 mg/kg q12 h or 2 mg/kg q24 h (max 60 mg/day)	Up to 2–6 weeks	*Taper:* over 2–6 weeks
Biologicals	**Anakinra** (recombinant interleukin-1 receptor antagonist)	SC	start at 2–3 mg/kg q12 h (max. 100 mg/dose)	Discuss in MDT	*Escalation/taper:* MDT decision. IV administration possible under different dosing scheme. *Side effects:* neutropenia, leukopenia, thrombocytopenia, eosinophilia, headache, abdominal pain, nausea/vomiting, diarrhea, hepatitis, increased serum transaminases, hypersensitivity reactions, injection-site reactions, skin rash, arthralgia
	**Tocilizumab** (recombinant interleukin-6 receptor)	IV	<30 kg: 12 mg/kg single dose (max 800 mg) ≥30 kg: 8 mg/kg single dose (max 800 mg)	Discuss in MDT	*Escalation:* If no clinical improvement after initial dose, may repeat dose 8–12 h after the initial dose after MDT discussion. *Side effects:* neutropenia, leukopenia, thrombocytopenia, anemia, pain, headache, dizziness, insomnia, demyelinating disorders, ulcerations, nausea, increased serum transaminases, liver impairment, increase in serum lipids, pancreatitis, hypertension, hypothyroidism, hypersensitivity reactions, Steven-Johnson-Syndrome, conjunctivitis, nephrolithiasis, injection-site reactions, rash
	**Infliximab** (chimeric tumor necrosis factor TNF α monoclonal antibody)	IV	5 mg/kg single dose	Discuss in MDT	*Side effects:* neutropenia, leukopenia/agranulocytosis, thrombocytopenia, anemia, pain, headache, dizziness, insomnia, demyelinating disorders, hypersensitivity reactions, injection-site reactions, skin rash

*Data were accessed from www.accessdata.fda.gov/, www.ema.europa.eu/, and the British Pediatric Allergy, Immunity, and Infection Group [Position Statement: Management of novel coronavirus (SARS-CoV-2) infection in pediatric patients in the UK and Ireland] and adapted from the Imperial College Healthcare NHS Trust PIMS-TS guideline for external use. MDT, multidisciplinary team; IVIG, intravenous immunoglobulins*.

*Disclaimer: Medication dosing and administration should be checked with the local hospital pharmacists and considering recent evidence updates. Where possible, PIMS-TS patients should be enrolled in interventional studies*.

For children with Kawasaki disease-like PIMS-TS and for children with coronary artery abnormalities irrespective of the PIMS-TS phenotype, the panel recommends that established institutional or international guidelines such as the 2020 American College of Rheumatology guidelines (40) on Kawasaki disease-like PIMS-TS should be followed. In addition, both the European SHARE initiative (41) and the American Heart Association guidelines (30) provide guidance on KD.

We recommend, based on currently available reports and in line with other recommendations, to use intravenous immunoglobulin (IVIG) in PIMS-TS patients presenting with shock, and to consider IVIG in PIMS-TS with undefined presentation. While IVIG should be usually administered as a single dose of 2 g/kg (max. 100 g/dose), clinicians should assess the cardiac and fluid status, particularly in patients in shock, as in some patients a slower administration become necessary.

While corticosteroids have often been used as adjuncts to IVIG (42), the specific indication, dosing, timing or type of glucocorticoids in PIMS-TS remains unknown. A French propensity-matched study of 111 children with PIMS-TS reported substantially improved outcomes in children with PIMS-TS treated with IVIG and steroids (9% persistence of fever) in comparison to children who received IVIG alone (51% treatment failure, Odd's ratio 0.25; 95%-CI 0.09–0.70, *p*-value 0.008) (43). The panel considered that benefit vs. harm justify pulse high-dose steroids for a duration of 1–3 days in PIMS-TS patients with shock, with an initial dose of 10 mg/kg methylprednisolone (max 1 g/day). Increasing the methylprednisolone dose to 30 mg/kg q24 h may be considered, although side effects such as hyperglycemia, hypertension, agitation, hospital-acquired infection, and hip osteonecrosis should be weighed against potential benefit. PIMS-TS patients treated with pulse corticosteroids should receive gastric protection with proton-pump inhibitors.

Given potential side effects and the lack of data, we advise against the use of pulse steroids in PIMS-TS patients without shock but suggest considering a lower dose therapy given intravenously (up to 2 mg/kg/day) and, subsequently, orally. While the optimal duration of steroid therapy in PIMS-TS remains unknown, the joint view of the panel is that decisions on steroid treatment duration should be guided by the clinical response (resolution of signs and symptoms), as well as by laboratory evidence of decreasing inflammation and improving organ function. Most patients seem to recover well under a 14-day treatment schedule without subsequent signs of disease rebound. In patients with apparent rebound, alternative inflammatory diagnoses should be considered.

A proportion of patients may not respond to initial treatment, and deterioration of the disease can be life-threatening. Refractory PIMS-TS is characterized by persistent fever and/or increase of inflammatory markers, worsening organ function, or increase in need of vasoactive drugs [measured by Vasoactive-Inotrope Score (44)] within 24–48 h after start of treatment. Consideration of disease severity in particular in PICU patients, and assessing evidence of persistent or progressive (multi-) organ dysfunction is paramount to guide treatment escalation.

Again, MDT assessment is recommended and needs to take differential diagnoses into account at every step before escalating treatment, and should carefully evaluate risk-vs.-benefit ratio of escalating immunomodulatory therapies. For example, PIMS-TS patients may develop secondary infections and immuno-suppressive treatment regimens may further impair the host defense against infection. The current list of biological drugs includes IL-1R (i.e., anakinra), IL-6R (i.e., tocilizumab) and tumor necrosis factor (TNF) (infliximab) blocking agents (Table 3). In addition, clinicians may discuss administration of a second dose of IVIG, or pulse steroids.

10. In patients with PIMS-TS refractory to initial treatment with IVIG and steroids, and after exclusion of alternative causes by the multidisciplinary team, we suggest consideration for anakinra. We recommend starting at 2–3 mg/kg q12 h s.c. (max. 100 mg/dose, total of 4–6 mg/kg/day). In case of clinical improvement, stopping of anakinra after 48–72 h should be considered in the multidisciplinary team.
11. In patients with PIMS-TS where no clinical and biochemical improvement to anakinra treatment is observed within 24–48 h, the multidisciplinary team should consider other targeted immunomodulation therapy with either tocilizumab or infliximab.
12. Clinical assessment and serial laboratory testing on measures of inflammation and organ dysfunction should guide escalation and duration of immunomodulation therapy treatment.

Despite its limited licensed indication, anakinra is increasingly chosen for off-label use in PIMS-TS patients (4, 45–47). Anakinra has been approved for subcutaneous administration, however continuous intravenous administration has been reported (48). Its short half-life (4–6 h) (Table 3), allows for repeated re-assessment of the immunosuppressive regimen. We therefore suggest a short trial of IL-1R blockade in PICU-hospitalized PIMS-TS patients that have not responded within a period of 24–36 h following administration of both IVIG and steroids. Anakinra dose increase in the absence of clinical improvement may be considered in the MDT. We suggest stopping anakinra after 48–72 h without tapering in case of clinical improvement. There is no evidence to support routine serial cytokine level assessment to guide cytokine-targeted therapy (CTT) (49).

Tocilizumab is an IL-6R blocking agent currently approved for the treatment of both systemic and polyarticular juvenile idiopathic arthritis in children above 2 years of age. Tocilizumab has also been used in children with PIMS-TS as IL-6 has been described to be one of the main drivers of the inflammatory cytokine storm in this disease entity. However, safety concerns exist as significant side effects have been described such as a reversible elevation of liver enzymes and an increased risk for bacterial and fungal infections (Table 3). Therefore, tocilizumab should be reserved for children with life-threatening PIMS-TS in whom anakinra has failed to show a clear benefit within 48 h. Given the long half-life (150 h) tocilizumab is usually given as a single intravenous dose.

The TNF-alpha blocking agent infliximab, classically used in children with various autoimmune inflammatory diseases has also been successfully used in children with KD. However, since infliximab increases the risk for secondary infections, and based on its long half-life (around 8 days, Table 3), infliximab should be administered as a single intravenous dose, and only in patients with PIMS-TS who failed to respond to anakinra (or tocilizumab).

### Anti-infective Therapies

13. Based on the absence of evidence of remdesivir treatment for children with COVID-19 and the proposed post-infectious concept of PIMS-TS (50) we do not recommend the routine administration of remdesivir in PIMS-TS patients.
14. Children with suspected PIMS-TS and signs of shock or other organ dysfunction should be treated empirically with intravenous broad-spectrum antimicrobial therapy for bacterial sepsis.
15. In children with PIMS-TS receiving intravenous antimicrobial therapy, we recommend daily assessment (e.g., clinical, laboratory assessment) for de-escalation of antimicrobial therapy in consultation with infectious diseases specialists. Stopping of antimicrobial therapy should be considered depending on the clinical course, microbiological findings, and the presence of PIMS-TS diagnostic criteria, including evidence of recent or current SARS-CoV-2 infection by serology or PCR.

Remdesivir, a nucleoside analog prodrug, has been approved for the treatment of COVID-19 in adults based on emerging evidence demonstrating the efficacy in shortening time to clinical recovery (51, 52), however recent experience (51, 53) has not confirmed these findings. The safety and effectiveness of remdesivir for treatment of COVID-19 in children has not yet been evaluated (studies are underway) outside case reports (54, 55). Hence the role of remdesivir in the management of PIMS-TS is uncertain, especially as PIMS-TS represents a post-infectious disorder rather than active SARS-CoV-2 infection. For these reasons the panel agreed that remdesivir should not be *routinely* used in children with PIMS-TS. However, PIMS-TS patients may be considered for the compassionate use of remdesivir on a case-by-case basis (31, 56). When remdesivir is used the FDA emergency use authorization instructions for the use of remdesivir in children >3.5 kg should be followed (57).

Children with PIMS-TS initially often present with signs and symptoms that mimic those of septic shock and toxic shock syndrome (34) and neither clinical findings (fever, rash, abdominal symptoms), infection markers (CRP), nor other laboratory measures of inflammation may allow reliable discrimination (58, 59). Mortality in children with sepsis and septic shock increases as time to effective antimicrobial therapy increases (60, 61). All suspected PIMS-TS patients with clinical signs of sepsis should therefore receive prompt empiric intravenous antibiotics within 1 h of presentation for those with shock, and within up to 3 h for those without shock (60). The choice of antibiotics should be based on local guidelines and taking into account age, epidemiology, signs consistent with toxic shock, and pre-existing medical conditions. Antibiotics should be stream-lined or stopped on the basis of the clinical course and microbiological culture results in discussion with the infectious diseases team.

### Supportive Measures

16. All PIMS-TS patients with shock or other organ dysfunction must be transferred to a center with availability of specialized Pediatric Intensive Care Units, cardiology, infectious diseases, and immunology/rheumatology.
17. Hemodynamic, respiratory, and other organ support should follow established guidelines such as the Surviving Sepsis Campaign.
18. Extracorporeal Membrane Oxygenation should be considered in PIMS-TS with cardiac, respiratory, or cardiorespiratory failure refractory to conventional management as per established guidelines such as the Surviving Sepsis Campaign.

Many PIMS-TS patients do not show evidence of active viral infection and may be less likely to be infectious to healthcare workers. However, clinical staff should wear appropriate personal protective equipment (31) as per institutional guidelines. Children should be triaged, assessed and management in line with recommendations for management of fever in infants <36 months, Surviving Sepsis Campaign (60), and standard Pediatric Advanced Life Support resuscitation algorithms for critically ill children. As some children's condition may worsen rapidly and they may develop hemodynamic compromise, close monitoring and early referral to a center with expertise in pediatric cardiology and PICU is mandatory. During resuscitation with fluid boluses, repeated assessment for signs of fluid overload is warranted (58) given risks associated with fluid overload, and considering that myocardial involvement is often seen in PIMS-TS.

19. In the absence of contraindications, we recommend starting prophylactic i.v. unfractionated heparin at a dose of 10 U/kg/h in PIMS-TS patients with shock. Conversion to low molecular weight heparin (LMWH, such as enoxaparin at a dose of 0.5 mg/kg q12 h) after the first day should be considered, depending on renal function.
20. In any other PIMS-TS patient requiring intensive care admission, we recommend prophylactic heparin (at a dose of 10 U/kg/h) or low molecular weight heparin (LMWH, such as enoxaparin at a dose of 0.5 mg/kg s.c. q12 h) depending on renal function.
21. For PIMS-TS patients not requiring PICU care, individual risk factors for thrombotic complications should be assessed to guide decisions on individualized anticoagulation therapy.
22. In all patients with coronary artery abnormalities antiplatelet management should follow established guidelines for Kawasaki disease.
23. As all patients with PIMS-TS irrespective of their phenotype are at risk for coronary artery aneurysms it is reasonable to administer low-dose acetyl salicylic acid (3–5 mg/kg, max. 100 mg) in all patients at least for 4–6 weeks until coronary abnormalities have been ruled out.

While there are only scarce data on the use of anticoagulation in children with PIMS-TS (40), there seems to be an increased risk of thromboembolic complications in patients infected with COVID-19, and as well during acute PIMS-TS (17, 62). These risks may potentiate the baseline risk of thromboembolic complications in critically ill children exposed to central venous devices, immobility, and other procedures (63). Therefore, the panel suggested to use prophylactic anticoagulation in those patients of higher severity requiring PICU admission. Clinicians should always weigh benefit of antiplatelet and anticoagulation treatments against the individual risk for clinically relevant bleeding. In acutely critically ill patients with shock the clinical course may rapidly change. For this reason we suggest starting with heparin rather than LMWH to enable stopping or reversal if clinically relevant bleeding occurs, and to avoid accumulation if renal function deteriorates.

In non-PICU patients, the overall assessment of the individual risk profile should include well-established risk factors such as a previous history of venous thromboembolism or a first-degree relative with venous thromboembolism, the presence of a central line, post-pubertal age or estrogen therapy amongst other. In addition, obesity may increase risk for thromboembolic events in patients with SARS-CoV-2 infection or PIMS-TS.

Similar to classical KD patients, children with PIMS-TS irrespective of the phenotype may be at risk for the development of coronary artery aneurysms. Hence it appears to be reasonable to consider low dose aspirin (3–5 mg/kg, maximum 100 mg, q24 h) in addition to prophylactic anticoagulation in all patients with PIMS-TS.

## Discharge From Hospital and Proposed Follow-Up

24. Patients who have been clinically well on regular wards with evidence of normal or recovering cardiac function, and who have been afebrile (<38 degrees) for 48 h should be considered for discharge home after MDT review.
25. A multi-disciplinary follow up is recommended for all patients at least after 1–2 weeks, and 4–6 weeks, respectively, post discharge from hospital. Follow-up should include pediatric immunology/rheumatology and cardiology, and, depending on the presentation and severity, other disciplines.
26. In case of cardiac involvement, cardiac follow up should be performed at least at 3, 6, and 12 months after discharge (Table 4).

**Table 4:** Proposed recommendations for clinical follow-up of PIMS-TS patients post-discharge.

	**Time since hospital discharge**						
**Initial cardiac involvement (at diagnosis or during hospitalization)**	**1–2 weeks**	**4–6 weeks**	**3 months**	**6 months**	**12 months**	**Exercise restriction^a^**	**General notes**
(1) No cardiac involvement	Echo, ECG, BP Consider 24 h-ECG^b^ Labs^c^ ASS	Echo, ECG, BP Consider 24 h-ECG^b^ Labs^c^ ASS = > consider stopping if no coronary artery abnormalities	Consider stopping follow-up at 4–6 weeks in patients without Kawasaki-like presentation; If coronary abnormalities at any time during follow-up = > follow AHA KD guidelines 2017 [see (5) below]			2 weeks	Consider involvement of other specialities if initial and/or persistent organ system abnormalities No live vaccines for 11 months following high-dose IVIG (2 g/kg)
(2) Myocardial injury (elevated cTnc ± NT-pro-BNP), normal ventricular function and normal coronary arteries or (3) Myocardial injury with initially depressed ventricular function, normalized systolic function at hospital discharge (diastolic dysfunction may persist)	Echo, ECG, BP Consider 24 h-ECG^b^ Labs^c^ ASS	Echo, ECG, BP Consider 24-h-ECG^b^ Labs^c^ ASS = > consider stopping if no coronary abnormalities	Echo, ECG, BP 24 h-ECG^a^ Exercise stress test^a^ (Labs^c^) Consider cardiac MRI^d^	Echo, ECG, BP Consider 24 h-ECG^b^ (Labs^c^)	Echo, ECG, BP Consider 24 h-ECG^b^ (Labs^c^)	3–6 months	
(4) Myocardial injury with persistent depressed ventricular function at hospital discharge	Echo, ECG, BP Consider 24 h-ECG^b^ Labs^c^ ASS	Echo, ECG, BP Consider 24 h-ECG^b^ Labs^c^ ASS = > consider stopping if no coronary abnormalities	Follow-up and sport restriction should be tailored based on the severity of the cardiac involvement and in line with guidelines for heart failure and myocarditis in children.				
(5) Coronary artery involvement	In addition to recommendations (1)–(4) above AHA guidelines 2017 should be respected with regards to follow-up, antiplatelet therapy/anticoagulation and exercise restriction.						

*ASS, acetylsalicylic acid; BP, blood pressure; CRP, C-reactive protein; Echo, echocardiography*.

a *24 h-ECG and exercise stress test should be performed before returning to sports activity*.

b *Consider 24 h-ECG if symptoms of arrythmia or abnormal ECG*.

c *Laboratory investigations should include FBC, inflammatory markers (CRP) and cardiac enzymes (CK, CK-MB, cTnC, NT-pro-BNP) as well as other values not normalized at hospital discharge (such as coagulation, U/E, LFTs). Also consider performing urine testing if previously abnormal or signs of renal abnormalities*.

d *MRI is considered in older patients without need for general anesthesia at 2–6 months post-discharge*.

Hospitals who do not have access to pediatric immunology, cardiology, or intensive care services should consult with the respective centers. In all children with PIMS-TS irrespective of the phenotype, consultation with cardiology is recommended before discharge to guide frequency of follow-up echocardiographic assessment, as cardiac function may not have fully normalized by the time of discharge and aneurysms may develop later in the course of the disease, even post-discharge. In general, we suggest performing a follow-up visit 1–2 weeks, and 4–6 weeks, respectively, after discharge which should encompass a multidisciplinary consultation. The visits should serve as well to critically review the indication, dosing, and duration of ongoing immunosuppressive therapy as appropriate. There is no evidence on duration and tapering schedules of steroids in PIMS-TS, nor on the recommended duration of hospitalization. Decisions on post-discharge treatment thus should be guided by ongoing laboratory or clinical evidence for persistent inflammation.

In children with cardiac manifestations, extension of the follow-up is recommended, and specific cardiac follow up is dependent on the degree of initial cardiac involvement, persistent myocardial dysfunction, coronary artery abnormalities, or rhythm disturbances. For all patients with coronary artery abnormalities a cardiologic follow up according to the American Heart Association KD guidelines is recommended (30). Hence, careful follow up should be considered to monitor myocardial function and coronary artery changes. Table 4 provides a suggestion on follow-up but it is important to acknowledge the lack of data to inform best follow-up practice at the present stage.

Exercise restriction is recommended for 2 weeks from discharge if there is no cardiac involvement; for 3–6 months if cardiac involvement was documented. ECG, echocardiography, exercise stress test and Holter monitor should be performed before resuming sport activity and cTnT and NT-pro-BNP should be normalized (27).

## Discussion

Within months of the COVID-19 pandemic spread, many countries across the globe have reported children presenting unwell with features of severe inflammation and multisystem disease (64, 65). Using available literature and guidelines from international institutions, the Swiss PIMS-TS recommendations represent best practice guidelines based on currently available knowledge to facilitate and standardize treatment of children with suspected PIMS-TS in Switzerland. The available literature is limited to retrospective and prospective studies from different part of the world (66, 67). There are no trials comparing different treatment modalities. The guidance on using various immunomodulatory agents is therefore limited to data from these observational cohort studies and indirect evidence from other hyperinflammatory conditions in children and adults.

These recommendation were established in exchange with experts from the U.K. and, accordingly, leverage from the RCPCH case definition. In comparison to another recently published institutional protocol on PIMS-TS from New York (26), our algorithm stratifies by severity defined as shocked/non-shocked rather than using specific vasopressor thresholds which are usually not applicable outside the intensive care environment. For children without shock presenting with undifferentiated PIMS-TS presentations, IVIG should be considered by the MDT team.

A number of limitations need to be considered. First, while the expert group includes specialists from the relevant disciplines, numbers of children with PIMS-TS during the first wave of COVID-19 in Switzerland were low, limiting experience in managing the disease (14, 45). However, the group assessed institutional pathways from other health care systems and consulted world leading experts in the field during the process. Second, the literature review performed was not systematic but focused. Third, to date there are no published results from randomized controlled trials in the field, and the evidence base for optimal PIMS-TS management remains minimal. Finally, recommendations were issued in the context of a well-resourced setting, where IVIG and biologicals are relatively easily available, and may not be applicable to resource limited settings. In relation to applying these guidelines, clinicians should be mindful of the risk of anchoring bias during the pandemic. Many children may test positive for COVID-19, not necessarily implying causality. The CDC, WHO and RCPCH case definitions of PIMS-TS bear a risk to over diagnose an assumedly rare syndrome in children who suffer from other common infectious or inflammatory or conditions such as septic shock, or rarer conditions such as HLH.

In conclusion, it is imperative that children with PIMS-TS are enrolled in prospective trials where feasible (68–70), and that clinical data are collected and shared to improve our understanding of the disease and its best management. Given the absence of high-grade evidence (20), regular revisions of these recommendations will be required when more evidence becomes available.

## Author Contributions

LS and PR designed and coordinated the work, wrote the first draft, and take responsibility for the content of the work. MA, NS, SG, and NR led subgroups and wrote the first draft on their section. All other authors participated in literature review, voting, writing, and have seen and approved the final version.

## Conflict of Interest

The authors declare that the research was conducted in the absence of any commercial or financial relationships that could be construed as a potential conflict of interest.
