# Supplementary material for: Best Practice Recommendations for the Diagnosis and Management of Children With Pediatric Inflammatory Multisystem Syndrome Temporally Associated With SARS-CoV-2 (PIMS-TS; Multisystem Inflammatory Syndrome in Children, MIS-C) in Switzerland
Source: Front Pediatr. 2021 May 26;9:667507. doi: 10.3389/fped.2021.667507 (PMC8187755; doi:10.3389/fped.2021.667507)
Supplement: Supplementary file 1 [file Data_Sheet_1.pdf]

**SUPPLEMENTARY MATERIAL FOR**

# Best Practice Recommendations for the Diagnosis and Management of Children with Paediatric Inflammatory Multisystem Syndrome Temporally Associated with SARS-CoV-2 (PIMS-TS; Multisystem Inflammatory Syndrome in Children, MIS-C) in Switzerland

***Authors***

Luregn J Schlapbach[1,2], MD, PhD, FCICM, Maya C Andre[3,4], MD, PhD, Serge Grazioli[5], MD, Nina Schöbi, MD[6,7], Nicole Ritz[8], MD, Christoph Aebi[6], MD, Philipp Agyeman[6], MD, Manuela Albisetti[9], Douggl G N Bailey[10], MD, Christoph Berger[11], MD, Géraldine Blanchard Rohner[12], MD, DPhil, Sabrina Bressieux-Degueldre[13], MD, Michael Hofer[12,14], MD, Arnaud G L'Huillier[12], MD, Mark Marston[3], MScN, Patrick M Meyer-Sauteur[11], MD, PhD Jana Pachlopnik Schmid[15], MD, PhD, Marie-Helene Perez[16], MD, Bjarte Rogdo[10], MD, Johannes Trück[11,15], MD, DPhil, Andreas Woerner[17], MD, Daniela Wuetz[18], MD, Petra Zimmermann[19,20], MD, PhD, Michael Levin[21,22], MD, PhD, Elizabeth Whittaker [21.22] MD, PhD, Peter C Rimensberger[5] MD, *for the PIMS-TS working group of the Interest Group for Pediatric and Neonatal Intensive Care (IGPNI) of the Swiss Society of Intensive Care and the Pediatric Infectious Diseases Group Switzerland (PIGS)*

***Corresponding author/contact:***

Prof. Luregn Schlapbach, MD, PhD, FCICM
Head, Pediatric and Neonatal Intensive Care Unit
University Children's Hospital Zurich – Eleonore Foundation
Steinwiesstrasse 75
CH-8032 Zurich Switzerland
phone +41 44 266 71 11
email: luregn.schlapbach@kispi.uzh.ch

**Appendix:**

**2005 International Pediatric Sepsis Definition Consensus Conference criteria for shock** (*as per Goldstein, B., et al. 2005 "International pediatric sepsis consensus conference: definitions for sepsis and organ dysfunction in pediatrics." Pediatr Crit Care Med 2005; 6(1): 2-8; with corrigenda in Gebara, B. M. et al. "Values for systolic blood pressure." Pediatr Crit Care Med 2005; 6(4): 500; author reply 500-501*).

Presence of the following despite appropriate intravenous fluid resuscitation:

- blood pressure <5th centile for age or systolic blood pressure <2 SD below normal for age

| *Age Group* | *Systolic Blood Pressure (mmHg)* |
|---|---|
| 0 days – 1 week | <59 |
| 1 week to 1 months | <79 |
| 1 months – 1 year | <75 |
| 1 – 5 years | <74 |
| 6-12 years | <83 |
| 13 - <18 years | <90 |

AND/OR

- need for vasoactive drugs to maintain blood pressure in normal range

AND/OR

- two of the following:
  - unexplained metabolic acidosis (base deficit >5.0 mEq/L)
  - arterial lactate >2 times upper limit of normal
  - oliguria (urine output <0.5 ml/kg/h)
  - capillary refill time >5 sec
  - core to peripheral temperature gap >3° Celsius

**HLH diagnostic criteria:**
*(as per Pachlopnik Schmid J, Volkmer B, Ehl S: "Classification, clinical manifestation and diagnosis of HLH". Abla O. and Janka G. (eds.): Histiocytic Disorders. Springer Verlag, Stuttgart 2018; page 173 – 187. ISBN (online): 978-3-319-59632-7)*

The diagnosis of HLH can be established if **(A)** and **(B)** are fulfilled
**A.** A molecular diagnosis consistent with HLH: disease-causing mutations in *PRF1*, *UNC13D*, *Munc18-2*, *STX11*, *RAB27A, LYST, SH2D1A*, or *BIRC4*

**B.** Five out of the eight criteria listed below are fulfilled:

1. Fever ≥ 38.5 °C
2. Splenomegaly (palpable below costal margin or increased size by imaging)
3. Cytopenia (affecting ≥ 2 out of the 3 lineages):
   Hemoglobin (<90 g/l; in newborns, <100 g/l)
   Neutrophilic granulocytes (<1.0 G/l)
   Platelet count (<100 G/l)
4. Hemophagocytosis (in the bone marrow or CSF)
5. Hyperferritinemia (≥ 500 µg/l)
6. Hypertriglyceridemia (fasting level, ≥ 3.0 mmol/l) or hypofibrinogenemia (≤ 1.5 g/l)
7. Elevated soluble CD25 (=soluble IL2 receptor, sIL2R) (≥ 2400 U/ml)
8. Decreased NK-cell cytotoxicity
